# Early-onset colorectal cancer patients without family history are “at very low risk” for lynch syndrome

**DOI:** 10.1186/1756-9966-33-1

**Published:** 2014-01-02

**Authors:** Vittoria Stigliano, Lupe Sanchez-Mete, Aline Martayan, Maria Diodoro, Beatrice Casini, Isabella Sperduti, Marcello Anti

**Affiliations:** 1 Division of Gastroenterology and Digestive Endoscopy, Regina Elena National Cancer Institute, IFO Via Elio Chianesi 53, 00144 Rome, Italy; 2 Division of Clinical Pathology, Regina Elena National Cancer Institute, IFO Via Elio Chianesi 53, 00144 Rome, Italy; 3 Division of Pathology, Regina Elena National Cancer Institute, IFO Via Elio Chianesi 53, 00144 Rome, Italy; 4 Division of Biostatistics, Regina Elena National Cancer Institute, IFO Via Elio Chianesi 53, 00144 Rome, Italy

**Keywords:** Early-onset colorectal cancer, Lynch syndrome, Immunohistochemistry, Microsatellite instability

## Abstract

**Introduction:**

Several studies evaluated the prevalence of Lynch Syndrome (LS) in young onset colorectal cancer (CRC) patients and the results were extremely variable (5%-20%). Immunohistochemistry (IHC) for MMR proteins and/or MSI analysis are screening tests that are done, either by themselves or in conjunction, on colon cancer tissue to identify individuals at risk for LS. The primary aim of our study was to evaluate the prevalence of LS in a large series of early-onset CRC without family history compared with those with family history. The secondary aim was to assess the diagnostic accuracy of IHC and MSI analysis as pre-screening tools for LS.

**Methods:**

Early-onset CRC patients (≤ 50 years) were prospectively recruited in the study. IHC and MSI analysis were performed in all the patients. Germ-line mutation analysis (GMA) was carried out in all MMR deficient tumors. A logistic regression model was performed to identify clinical features predictive of MSI-H.

**Results:**

117 early onset CRC cases were categorized in three groups (A, B, C) according with family history of CRC. IHC and MSI analysis showed MMR deficiency in 6/70 patients (8.6%) of group A, 24/40 patients (60%) of group B and none of group C. GMA showed a deleterious mutation in 19 (47.5%) patients of group B. MSI analysis had a diagnostic accuracy of 95.7% (CI 92.1-99.4) and IHC of 83.8% (CI 77.1-90.4). The logistic regression model revealed that by using a combination of the two features “No Amsterdam Criteria” and ”left sided CRC” to exclude MSI-H, accuracy was 89.7% (84.2-95.2).

**Conclusions:**

Early-onset CRC patients, with left sided CRC and without family history are “at very low risk” for Lynch syndrome. The two simple criteria of family history and CRC site could be used as a pre-screening tool to evaluate whether or not patients should undergo tissue molecular screening. In the few cases of suspected LS (right sided CRC and/or Amsterdam Criteria), a reasonable approach could be to perform MSI analysis first and IHC afterwards only in MSI-H patients.

## Introduction

The Lynch Syndrome (LS) is an autosomal dominant condition with incomplete penetrance, predisposing to colorectal cancer (CRC) and other malignancies at a young age due to a germline mutation in one of the Mismatch Repair (MMR) genes (MLH1, MSH2, MSH6 and PMS2) [1-3]. CRC of patients with Lynch syndrome shows MMR deficiency, defined by the presence of microsatellite instability (MSI) and loss of the MMR protein expression, which is the hallmark of this disorder [3]. The syndrome accounts for 2%–4% of all CRCs and the lifetime risk of developing CRC in the MMR mutation carriers is estimated to be 50%–80% [4,5]. Therefore, patients with LS and their relatives have to undergo intensive surveillance and appropriate management to improve their survival [6-8].

The most widely used diagnostic strategy for Lynch syndrome is based on selecting patients who fulfil the Amsterdam criteria [2] or any of the Revised Bethesda Guidelines [9], followed by Tumour (Tissue) Testing of MSI and/or immunostaining (IHC) of MMR proteins and germline mutation analysis in MMR deficient cases. The Amsterdam Criteria allow to select patients on the basis of familial segregation and early age at onset of CRC or other cancer in LS spectrum. The Revised Bethesda Guidelines are less stringent and consider age at onset, presence of synchronous/metachronous cancer (multiple primary cancer), MSI-H phenotype at age < 60 years and familial history of cancer in LS spectrum separately. Both clinical criteria emphasize the importance of early age at onset (≤ 50 years) to suspect LS. Furthermore, recent findings suggest an increasing incidence of CRC in young patients [10-12] as well as the association with advanced stage, prevalent distal location and poor prognosis [10,13-19]. Therefore, patients with CRC at age ≤ 50 yrs have been considered for LS screening in several studies and the prevalence of LS in early onset-CRC cohorts resulted extremely variable accounting for about 5% to 20% [13,20-32]. The heterogeneity of the results of these studies is likely due to different methodological approaches, kind of cohort studied and different molecular strategies used for detecting LS.

The variability of molecular strategies reflects that, at present there is considerable uncertainty regarding whether to recommend IHC or MSI or the combination of both as a primary screening tool [33-35].

Some authors found a similar effectiveness of both techniques to screen LS, but consider IHC less complex and suggest to start with it [33]. The recent Jerusalem Workshop [34] recommended to use IHC or MSI alternatively, whereas the last revised NCCN guidelines [35] propose to use a combination of both as testing strategies for LS in high risk subjects.

The primary aim of our study was to evaluate the prevalence of Lynch syndrome in a single-center large series of early-onset CRC without family history compared with those with family history of CRC and/or other malignancies of LS spectrum.

The secondary aim was to evaluate sensitivity, specificity and predictive values of both tests to select patients for mutational analysis and identify LS in early onset CRC without family history.

## Methods

### Patient’s accrual

From January 2007 to December 2012, patients with a history of colorectal cancer (CRC) and age at diagnosis ≤ 50 years, who were referred to Hereditary CRC Clinic of Regina Elena National Cancer Institute, were prospectively recruited in the present study.

Patients with Familial Adenomatous Polyposis (FAP), Hyperplastic Polyposis, Hamartomatous Polyposis syndromes, MUTYH associated polyposis and inflammatory bowel disease were excluded from the study.

For each patient an informed consent form was signed and approved by the IFO Institutional Ethics Committee and personal medical history, detailed oncological family history were recorded and evaluated according to the Amsterdam II Criteria [35].

Immunohistochemistry for MMR proteins and microsatellite instability (MSI) analysis on tumour sampling were performed in all the patients. Tumors were considered MMR deficient if they were MSI-H and/or showed lack of MMR protein expression. Germline mutation analysis of MLH1 or MSH2 was carried out in all cases with a total lack of expression for MLH1 and no promoter hypermethylation or loss of MSH2 at immunohistochemistry, respectively. MSH6 genetic testing was done in patients whose tumor showed loss of MSH6 expression or a combined lack for MSH2 and MSH6 expression but did not have MSH2 mutations. Patients with a loss of MSH2 expression with no MSH2 or MSH6 mutations detected were analysed for EpCAM rearrangements. PMS2 genetic testing was performed in patients showing isolated loss of PMS2 expression or a combined lack of MLH1 and PMS2 expression but did not have MLH1 mutations. In patients with MSI-H tumor and normal or not available MMR protein expression, the four MMR genes were investigated in order of decreasing prevalence.

### Immunohistochemistry and microsatellite instability analysis

Tissues (surgical sample) from colorectal adenocarcinoma patients were collected and stored in the Institute’s Tissue Bank. Patients who did not undergo surgery at our Institution were asked to apply for pathological specimens/slides at the Pathology Unit of the Hospital in which they had surgery. The expression of MLH1, MSH2, MSH6, PMS2 genes was assessed by IHC on 2 micron thick sections of routinely formaline-fixed and paraffin-embedded blocks of selected colon adenocarcinoma tissues. Monoclonal antibodies BioCARE MEDICAL, MLH1 (clone G168-18), MSH2 (clone FE11), MSH6 (BC/44) and PMS2 (tipo clone A16-4) were used in an automated Bond immunostainer (Vision-Biosystem. Menarini, Florence, Italy). A pathologist with vast gastrointestinal experience scored the gene as expressed (positive) when nuclear staining in tumour tissue was present or, as not expressed (negative), when nuclear staining was absent.

Microsatellite instability was assessed on DNA extracted from microsections of paraffin-embedded blocks of selected colon adenocarcinoma tissues. The analysis was performed comparing the allelic profiles of microsatellite markers generated by amplification of DNA from matching normal and tumour samples. A panel of seven microsatellite markers (5 mononucleotide and 2 pentanucleotide repeats) was used (MSI Analysis system Version 1.2– Promega). Samples were run on an Applied Biosystems 3130 Genetic Analyzer (Life Technologies). Output data were analyzed with GeneMapper® Analysis Software (Life Technologies). MSI status was assigned as MSI high (MSI-H, ≥ 30% markers unstable), MSI low (MSI-L, < 30% markers unstable), or microsatellite stable (MSS, no unstable markers).

### Methylation analysis

MMR genes promoter methylation was investigated by Methylation-Specific MLPA (MS-MLPA) following the manufacturer’s instructions (SALSA MLPA kit ME011-B1) [36,37]. Methylation analysis was performed by comparing MMR gene promoter methylation profiles of tumour samples and that of normal adjacent tissue. PCR products were analyzed on an 8 capillary 3500 DX Genetic Analyser (Life Technologies) using GeneMapper v4.1 software (Life Technologies). A dosage ratio of 0.15 or higher, corresponding to 15% of methylated DNA, was interpreted to indicate promoter methylation.

### Mutation analysis

Four MMR genes were extensively analysed in our study: MLH1, MSH2, MHS6 and PMS2. The coding exons and exon-intron boundaries of each gene were amplified under optimized PCR conditions and directly sequenced. Primer sequences and PCR conditions are available upon request. MLPA reactions were performed following the manufacturer’s instructions (MRC-Holland, Netherlands), and the test kits used were SALSA MLPA P003, P008, P072 and P248. Since deletions of the most 3’ exon of EPCAM can result in silencing the MSH2 gene, this region was also analyzed (SALSA MLPA P003-B1 kit includes two probes for the most 3’ exon of EPCAM). If an aberrant MLPA result was observed, relative quantification with Real-Time PCR was performed as a confirmatory test (LightCycler480II – Roche). Genomic DNA and total RNA extractions were performed using respectively the QIAamp DNA blood Mini Kit (QIAGEN) and the RNeasy Plus mini Kit (QIAGEN). RT-PCR was performed using the SuperScript® One-Step RT-PCR System with Platinum®Taq DNA Polymerase (Life Technologies). Full-length sequencing was held on an 8 capillary 3500 DX Genetic Analyser (Life Technologies) and data was analysed with Mac Vector 9.0 ClustalW (v1.4) multiple sequence alignment software (Accelrys). MLPA data were analysed with Coffalyser Software. Classification of genomic variants was performed pooling the information reported in the publicly accessible InSiGHT database (International Society for Gastrointestinal Hereditary Tumours) and findings gathered from peer-reviewed journals and literature and other public genomic data sources. As to variants of unknown clinical significance and new variants, four sets of data were integrated. These were: (a) analysis of the segregation with the disease of the variant of interest in as many family members as possible; (b) analysis of pooled family histories of index cases carrying the same variant; (c) search for the co-occurrence between the unclassified variant of interest and a clearly deleterious variant in the same gene; and (d) assessment of the degree of evolutionary variation of the mutation of interest in a multiple sequence alignment.

### Statistical analysis

Pearson’s Chi-Square test or Fisher’s Exact test were used, when appropriate, to evaluate associations between the variables. The Odds Ratio (OR) and the 95% confidence intervals (95% CI) were estimated for each variable. A multivariate logistic regression model was also developed using stepwise regression (forward selection) to compare the predictive power for modulation of different factors. Enter limit and remove limit were p = 0.10 and p = 0.15, respectively. The assessment of interactions between significant investigation variables was taken into account when developing the multivariate model.

Multivariate models based on regression tree analysis were explored to establish the most discriminative combination of variables to identify MSI-H. Recursive partitioning programs build classification or regression models of a very general structure using a 2-stage procedure; the resulting models can be represented as binary trees.

Performance characteristics, accuracy, sensitivity, specificity, positive (PPV) and negative (NPV) predictive values and areas under the curves (AUC) were evaluated with respect to the presence of MSI–H on tumor specimen by computing Receiver Operating Characteristic (ROC) curves.

The SPSS®(20.0) statistical program was used for all the analyses.

## Results

### Patients

117 early onset CRC cases were recruited in the study and were categorized in three groups:

•Group A, 70 cases with CRC diagnosed at age ≤ 50 and no family history of CRC and/or other malignancies of LS spectrum.

•Group B, 40 cases with CRC diagnosed at age ≤ 50 and Amsterdam II Criteria fulfilled.

•Group C, 7 cases with CRC diagnosed at age ≤ 50 and family history of CRC, not fulfilling the Amsterdam II criteria.

The median age at diagnosis of CRC was 42 years (range 20–50 years) in group A, 45 years (range 28–50 years) in group B and 39 years in group C (range 36–46 years); gender distribution (male/female) was 26/44 in group A, 19/21 in group B and 3/4 in group C (p = 0.57).

16 out of 70 patients of group A (22.9%), 21 out of 40 of group B (52.5%) and 2 out of 7 (28.6%) patients of group C had a right-sided colorectal cancer (proximal to the splenic flexure) (p = 0.006). There was no significant difference in staging at diagnosis between the three groups: an advanced stage (III, IV) was present in 38 out of 70 pts from group A (54.3%) vs 17 out of 40 patients from group B (44.7%) and 4 out of 7 from group C (57.1%) (p = 0.61).

Multiple primary cancers (synchronous, methachronous, extracolonic) were not reported in group C, occurred more frequently in group B (12 patients, 30%) than in group A (4 patients, 5.7%) (p = <0,0001) and had the following distribution: an extracolonic cancer was present in 2 out of 70 patients in group A (2.9%) vs 10 out of 40 in group B (25%) (p = <0.0001) and the spectrum of extracolonic cancers was more heterogeneous in group B than in group A; metachronous cancers were recorded in 4 out of 70 patients (5.7%) in group A vs 10 out of 40 (25%) in group B (p = 0.007); synchronous cancers were found in 2 out of 70 patients (2.9%) in group A vs 6 out of 40 (15%) in group B (p = 0.04) (Table 1).

**Table 1:** Patient characteristics and comparative analysis of principal clinical features consistent with LS between the three groups

**Characteristic**	**No family history (group A, n = 70)**	**Am. II**^**§§ **^**criteria (group B, n = 40)**	**Family history without Am.II criteria (Group C, n = 7)**	**P-value**^**§**^
**Median age (years), range**	42 (20–50)	45 (28–50)	39 (36–46)	
**Gender distribution**				
** M**	29	18	3	
** F**	48	22	4	
**Right sided CRC (%)**	16 (22.9)	21 (52.5)	2	0,006
**Multiple primary cancer (%)**	4 (5.7)	12 (30)	0	<0.0001**
**Extracolonic cancer (%)**	2 (2.9) (thyroid, pancreas)	10 (25) (3 endometrium, 2 breast, 2 kidney, 1 stomach, 2 ovary, 3 sebaceous skin tumours)*	0	<0.001**
**Metachronous cancer (%)**	4 (5.7)	10 (25)	0	0.007**
**Synchronous cancer (%)**	2 (2.9)	6 (15)	0	0.04**

*4 cases were multiple primary cancer.

**AvsB.

^§^Fisher’s Exact test was used, to evaluate associations between the variables.

^§§^AM.II: Amsterdam II.

### Molecular genetic analysis

In group A, 64 out of 70 patients (91.4%) expressed all MMR genes at IHC and did not show the MSI-H phenotype. 6 out of 70 patients (8.6%) showed MMR deficiency: two had lack of expression of PMS2 and displayed MSI-H; three had lack of expression of MLH1/PMS2 and showed MSS; one had a normal expression of all MMR genes and showed MSI-H. Germline mutation analysis was performed in all six patients and no deleterious mutations were found. In one out of the three MSI-H patients, lacking PMS2 expression, the genetic testing revealed an hypermethylation of MLH1 promoter. In the other two MSI-H patients a polymorphism of MSH6 gene (c.116G > A; p.Gly39Glu; rs1042821) reported to be associated with a slight increased risk of CRC in males [38] was detected (Table 2).

**Table 2:** Results of molecular screening on tumor specimen and mutational analysis

**Patients**	**Immunohistochemistry (lack of expression)**	**MSI status**	**Germline mutational analysis**
**Group A**	1 PMS2	1 MSI-H	No deleterious mutation§
**No family history**	1 PMS2	1 MSI-H	No deleterious mutation*
	3 MLH1, PMS2	3 MSS	No deleterious mutation
	1 normal	1 MSI-H	No deleterious mutation*
**Group B with Am.II Criteria**	8 MLH1	8 MSI-H	7 MLH1 deleterious mutation
			1 missense VUS**
	7 MSH2	7 MSI-H	7 MSH2 deleterious mutation
	1 MSH2***	1 MSI-H	1 MLH1 deleterious mutation
	1 PMS2	1 MSI-H	1 MLH1 deleterious mutation
	2 Normal	2 MSI-H	2 MSH2 deleterious mutation
	1 NE****	1 MSI-H	1 MSH2 deleterious mutation
	1 MSH2, MSH6	1 MSI-H	No deleterious mutation
	4 MLH1	4 MSS	No deleterious mutation
	1 MLH1, PMS2	1 MSS	No deleterious mutation
	1 MSH2, MSH6	1 MSS	No deleterious mutation
**Group C**	7 normal	7 MSS	
**Family history without Amsterdam II Criteria**			

§MLH1 promoter hypermethylation.

*polymorphism MSH6 gene (c.116G > A) associated with slight increased risk of CRC in males.

**VUS: variant of uncertain clinical significance.

***confirmed after repeating the test.

****NE: not evaluable.

In group B, IHC showed MMR deficiency in 24 out of 40 patients (60%) and MSI –H in 21 (52.5%). Germline mutation analysis was performed in all 24 patients and a deleterious mutation in the corresponding IHC lacking protein was detected in 15 (62.5%), 8 in MLH1 gene and 7 MSH2, all these patients were MSI-H. IHC detected an altered expression of MSH2 in another MSI-H patient, whereas the deleterious mutation was found in MLH1. In the remaining 5 out of 21 MSI –H patients the germline mutation analysis revealed:

•A deleterious mutation in the MSH2 gene in three patients with normal or not assessable MMR expression at IHC.

•A missense variant of uncertain clinical significance of MLH1 gene: c.376 T > A. (p.Tyr126Asn) in one case with MLH1 altered expression at IHC. The available data on the clinical impact of this variant are so far not unequivocal [38].

•No deleterious mutation in the four MMR genes analyzed was found in one case with lack of expression of MSH2 at IHC.

•In Group C, IHC revealed normal expression of MMR protein and MSS in all patients (Table 2).

### Diagnostic accuracy of molecular screening tests and of clinical variables

**Figure 1:**
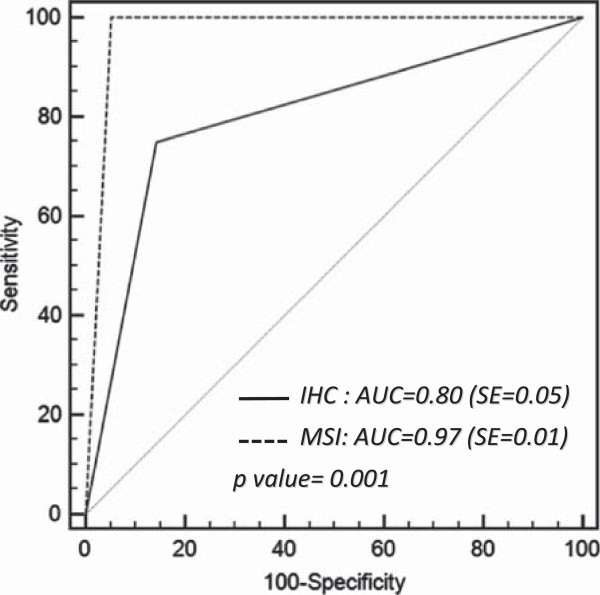
**ROC curve analysis of molecular screening tests.** The two ROC curves represent the diagnostic accuracy of Microsatellite Instability analysis (MSI) and Immunoistochemistry (IHC) to identify and select MMR deficient early onset colorectal cancer patients for mutational analysis. Accuracy is measured by the Area Under the Curve (AUC) and is significantly higher in MSI than IHC (AUC 0.97 vs 0.80, p = 0.001).

In our series, we observed the following diagnostic accuracy of molecular screening tests in predicting germline mutations of MMR genes: MSI analysis had a sensitivity of 100%, a specificity of 94.8% (CI 86.2-100) a diagnostic accuracy of 95.7% (CI 92.1-99.4), a PPV of 80% (CI 72.0-88.0), a NPV of 100% and an AUC of 0.97 (standard error, SE = 0.01); IHC had a sensitivity of 75% (IC 66.0-84.0), a specificity of 85,6% (CI 72.8-98.4) a diagnostic accuracy of 83.8% (CI 77.1-90.4), a PPV of 51.7% (CI 41.8-61.7), a NPV of 94.3% (CI 84.2-100) and an AUC of 0.80 (SE = 0.05) (Figure 1).

Considering the clinical variables gender, stage, cancer site and multiplicity, the presence of extracolonic cancers and Amsterdam II criteria, a logistic regression model was performed to evaluate the independent variables predictive of MSI-H phenotype in early onset CRC. The unique factors associated with MSI-H were Amsterdam II Criteria (P < 0.0001) and right-sided CRC (P < 0.0001). In fact, in the Amsterdam group we observed that 80.9% of right-sided vs 26.3% of left sided CRC were MSI-H (p = 0.0005) whereas in the subgroup without Amsterdam II criteria only, 11.1% of the right-sided vs 1.7% of the left sided CRC were MSI-H (p = 0.13).

**Figure 2:**
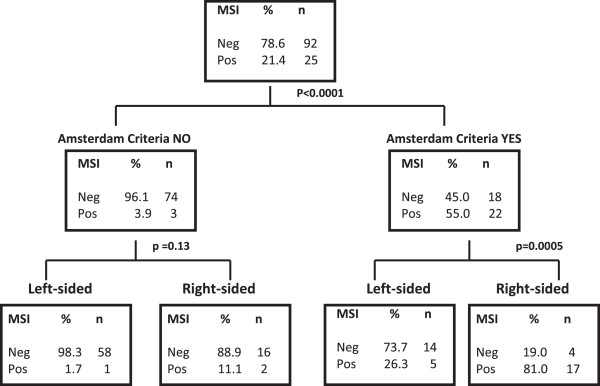
**Regression tree to evaluate the features predictive of MSI-H.** In the Amsterdam group 81% of right-sided vs 26.3% of left sided CRC were MSI-H (p = 0.0005) whereas in the subgroup without Amsterdam II criteria only 11.1% of the right-sided vs 1.7% of the left sided CRC were MSI-H (p = 0.13). To confirm and evaluate (analyze) these results, we built a Regression Tree which revealed that by using a combination of the two features “No Amsterdam Criteria” and “left sided CRC” to exclude MSI-H the accuracy was 89.7% (84.2-95.2).

To confirm these results, we built a Regression Tree which revealed that by using a combination of the two features “No Amsterdam Criteria” and “left sided CRC” to exclude MSI-H, accuracy was 89.7% (84.2-95.2) (Figure 2).

## Discussion

The present study aimed at evaluating whether early age at onset of CRC is a crucial risk factor for LS, apart from family history. Therefore, we selected a large subset of early-onset CRC and stratified patients according to the family history: Amsterdam II criteria fulfilled, family history of CRC without Amsterdam II criteria and no family history. Tissue molecular analysis on tumor specimen was performed in all the patients and germline mutation analysis was carried out in MMR deficient cases.

The main result of our study was that no LS affected patients were identified among the patients with no family history or one or more first degree relative. Among the 40 patients fulfilling Amsterdam II criteria, by contrast, 19 (47.5%) LS cases were diagnosed. These data are in agreement with those of Jasperson et al. [20] which reported a low frequency (6.5%) of MMR germline mutations among young patients without family history suspecting LS and found 73.3% of MMR germline mutations in the cases with Amsterdam Criteria. Other authors reported a highly variable prevalence of MMR gene mutation carriers in early onset CRC, ranging between 4.2% and 17.7% [13], [21], [23], [24], [26][27], [31], [32], [39], but the number of cases without family history was specified in few studies [21,27,31]. If we only consider these studies, we will observe a dramatic decrease in the LS prevalence rate to 3.5%-6.4%, in agreement with our results.

In our series, we observed that the principal clinical features consistent with LS (right-sided CRC, multiple primary, extra-colonic, synchronous or metachronous cancer) were significantly less represented in the group without having fulfilled Amsterdam criteria. In particular, in these two groups, the left colon was more frequently involved (77.1% of cases in group A and 71.4% in group C) (Table 1). Previous studies on young CRC series reported, as well, a predilection for the distal colon ranging from 55 to 80% of cases [4,11,21,23,27,29,31,32],[39,40]. The heterogeneity between these studies, compared to the CRC site in early onset CRC, maybe related to the proportion of LS detected in each study, as LS related CRC mainly occurs in the right colon. On this basis, we could consider two (different clinico-pathological) subsets of early onset CRC: the greatest percentage represented by left sided CRC without important family history (no Amsterdam Criteria fulfilled) and the lowest percentage represented by LS related CRC, with Amsterdam II criteria fulfilled and typical features of the syndrome. Our major concern was whether we should have performed a molecular screening in both subsets of early onset CRC. In order to address this issue and considering that all Lynch syndrome associated CRC display MSI-H [4], we performed a logistic regression model to identify features predictive of MSI-H. The regression tree revealed, indeed, that using the combination of the two features “No Amsterdam Criteria” and “left sided CRC” to exclude MSI-H, has an accuracy of 89.7% (Figure 2).

Interestingly, in the group with no family history, we identified 3 MSI-H cases. The germline mutation analysis did not confirm LS diagnosis in any of the patients as MMR deleterious mutations were not found. Despite this, we observed an acquired MLH1 promoter hypermethylation in one case, with loss of PMS2 expression at IHC. Lack of MLH1 expression affects PMS2 protein stability and explains its loss at IHC, thus we classified this case as “sporadic colorectal cancer” [41]. Moreover, we identified a single nucleotide polymorphism (c.116G > A; p.Gly39Glu; rs1042821) in the MSH6 gene, in two cases in which IHC detected a normal expression of the corresponding protein. This polymorphism (MSH6 G39E) encodes a non-conservative amino acid change where it is unknown whether the variant affects protein function. MSH6 G39E is reported, in one study to confer a slight risk of CRC in males (OR 1.27; 95% CI 1.04 to 1.54), higher in MSI-H than MSS (OR 1.30; CI 95%) [38]. Other authors reported in MSH6 G39E homozygous patients an increased risk of rectal cancer only [42]. The observed association should be interpreted with caution, since no association was found between the MSH6 variant and the overall CRC, probably due to the small number of rectal cases included in the study.

The secondary aim of the present study was to compare the diagnostic accuracy of IHC and MSI analysis in early onset CRC to select the best technique to start with in the suspected LS. We observed that MSI analysis had a higher diagnostic accuracy (95.7% vs 83.8%) sensitivity (100% vs 75%), specificity (94.8% vs 85.6%) and AUC (0.97 vs 0.80) than IHC (Figure 1). In fact, had we not used MSI analysis, we could have missed four LS cases not detected by IHC in the group with Amsterdam II Criteria. Even in the early-onset group, IHC was misleading as it showed a lack of expression of MMR genes in three MSS patients in which the germline mutation analysis did not reveal any deleterious mutation. The main factors potentially affecting IHC staining are tissue processing, antigen retrieval procedures, the type of fixative and duration/condition of tissue fixation [43,44]. Therefore, even if it allows the identification of the target gene for mutational analysis, IHC “sometimes” suffers from technical limitations and should be performed in combination with MSI analysis or afterwards.

Both techniques, IHC and MSI analysis, require a pathology laboratory and interpretation by experts. In clinical practice, we shall consider a cost effective algorithm and given the similar costs of the two methods the choice between them will depend on sensitivity and specificity of the test and on the local expertise. Our data suggest that Microsatellite instability analysis has a higher diagnostic accuracy than immunohistochemistry, therefore it should be worthwhile to perform it first and consider IHC staining only in the MSI-H selected cases.

## Conclusions

In conclusion, we can state that if we are dealing with an early-onset CRC patient, with left sided CRC and without family history, a diagnosis of LS is highly unlikely.

We could consider this subset of patients “at very low risk” for Lynch syndrome and can use the two simple criteria, family history and CRC site, as a pre-screening tool to evaluate whether or not patients should undergo tissue molecular screening. This approach will allow the physician to reduce unnecessary tests in the subset of patients “at very low risk for LS”. In the few cases of suspected LS (right sided CRC and/or Amsterdam Criteria), a reasonable approach could be to perform MSI analysis first and consider IHC staining only in the MSI-H patients.

Further studies are surely needed to clarify the carcinogenesis mechanism in the increasing number of cases of early onset CRC without LS.

## Competing interests

The authors declare that they have no competing interests.

## Authors’ contributions

VS conceived of the study, participated in its design and coordination and performed clinical and endoscopic examination. LSM collected data, performed clinical and endoscopic examination and drafted the manuscript. AM carried out the mutational analysis, MD and BC carried out immunohistochemistry and Microsatellite instability analysis, IS performed statistical analysis and MA provided a critical revision of the manuscript. All authors read and approved the final manuscript.

## Authors’ information

Dr Vittoria Stigliano is the director of the Hereditary CRC Clinic of Regina Elena National Cancer Institute.
